# Immediate Effects of a Pogo-Jump Microdose on Vertical Jump Performance in Adolescent Basketball Players: A Randomized Crossover Pilot Study

**DOI:** 10.3390/jfmk11030366

**Published:** 2026-09-10

**Authors:** Celia García-Lucas, Gemma Biviá-Roig, Ramón Pascual-Morales, Jorge Escribá-Fuentes, Iván Muñoz-Martínez, Sergio Samper-Llavador, Francesc Josep Llidó-Barreres, Pedro Múzquiz-Barberá, Juan Francisco Lisón, Juan José Amer-Cuenca

**Affiliations:** 1 Departamento de Ciencias Biomédicas, Facultad de Ciencias de la Salud, Universidad Cardenal Herrera-CEU, CEU Universities, C/Ramón y Cajal, s/n, 46115 Alfara del Patriarca, Spain; celia.garcialucas1@uchceu.es; 2 Departamento de Fisioterapia, Facultad de Ciencias de la Salud, Universidad Cardenal Herrera-CEU, CEU Universities, 46115 Alfara del Patriarca, Spain; gemma.bivia@uchceu.es (G.B.-R.); ramon.pascualmorales@alumnos.uchceu.es (R.P.-M.); jorge.escribafuentes@alumnos.uchceu.es (J.E.-F.); ivan.munozmartinez@alumnos.uchceu.es (I.M.-M.); sergio.samperllavador@alumnos.uchceu.es (S.S.-L.); francesc.llidobarreres@alumnos.uchceu.es (F.J.L.-B.); pedro.muzquizbarbera@uchceu.es (P.M.-B.); juanjoamer@uchceu.es (J.J.A.-C.); 3 Centre of Physiopathology of Obesity and Nutrition (CIBERobn), CB06/03 Instituto de Salud Carlos III, 28029 Madrid, Spain

**Keywords:** post-activation performance enhancement, pogo jumps, plyometric exercise, stretch-shortening cycle, countermovement jump, drop jump, adolescent athletes, basketball

## Abstract

**Background and Objectives:** Low-volume plyometric conditioning may elicit post-activation performance enhancement (PAPE), but the early response to pogo-jump exercise in adolescent basketball players remains uncertain. This randomized crossover pilot study estimated the effect of adding 24 bilateral pogo jumps to a standardized warm-up on countermovement-jump (CMJ) and drop-jump (DJ) height, compared with time-matched low-intensity walking. **Methods:** Twenty-seven male basketball players were recruited, and 21 (age, 16.55 ± 0.88 years) completed both conditions. In randomized order, separated by a 7-day washout, participants performed 3 sets of 8 bilateral pogo jumps with 1 min between sets or approximately 3 min of low-intensity walking. Post-condition jump assessments began after a 1 min recovery and comprised three consecutive CMJs, a 30 s transition, and three consecutive DJs, with all measurements completed within approximately 2 min after each condition. The highest valid trial was retained for each jump type. Period-adjusted treatment effects were estimated using separate linear mixed-effects models, with repeated-measures ANOVA and Wilcoxon analyses retained as sensitivity analyses. **Results:** The period-adjusted between-condition difference in change was −0.89 cm for CMJ (95% CI, −2.92 to 1.14; *p* = 0.389) and 0.34 cm for DJ (95% CI, −1.77 to 2.45; *p* = 0.753). Sensitivity analyses yielded the same overall interpretation. All completers performed the protocol, and perceived exertion was 11.4 ± 1.2. The pogo-jump protocol showed favorable immediate tolerability, with all completers reporting 0/10 anterior-knee pain immediately after the condition. **Conclusions:** The pilot estimates did not indicate a detectable group-level improvement in CMJ or DJ height during the early post-conditioning assessment. However, the confidence intervals were compatible with potentially meaningful effects in either direction. The small sample, single dose, single early assessment window, and variable DJ reliability limit the precision and generalizability of the findings.

## 1. Introduction

Basketball repeatedly exposes players to high-intensity accelerations, decelerations, changes in direction, and jumps. Vertical-jump capacity is relevant to basketball performance and is commonly used for field-based assessment of lower-limb neuromuscular function. The countermovement jump (CMJ) evaluates maximal vertical-jump performance during a relatively long stretch-shortening cycle, whereas the drop jump (DJ) imposes faster stretch-shortening-cycle demands and requires rapid force production after landing [1,2,3]. Although contact-time-derived variables are required to quantify reactive strength directly, CMJ and DJ height provide complementary information about performance in mechanically distinct jumping tasks.

Post-activation performance enhancement (PAPE) refers to an acute improvement in voluntary performance following a conditioning activity and should be distinguished from post-activation potentiation assessed through electrically evoked twitch responses [4]. The observable response reflects the net balance between performance-enhancing and fatiguing processes and is influenced by the conditioning activity, exercise volume, recovery interval, training status, and similarity between the conditioning and performance tasks. Recent systematic reviews indicate that conditioning activities can improve subsequent jump performance on average, while also highlighting substantial protocol-dependent and inter-individual variability and limitations in the certainty of the evidence [5,6].

Plyometric exercises are attractive conditioning activities because they can be performed with maximal intent, require little or no equipment, and resemble sport-specific explosive actions. Acute improvements in jump performance have been reported after plyometric conditioning activities, although previous protocols have generally used greater exercise volumes and several post-conditioning assessment times [7,8]. Pogo jumps constitute a more ankle-dominant alternative involving repeated bilateral hops with limited knee excursion and an emphasis on short ground-contact time. A low-volume bout could therefore provide a practical stimulus during warm-up or a brief interruption in play while limiting residual fatigue.

The timing of assessment is particularly relevant. Some plyometric conditioning studies have detected performance enhancement within the first minute, whereas meta-analytic evidence suggests that the response may peak several minutes later and varies with the protocol and athlete characteristics [5,7,8,9,10]. An assessment at 1 min therefore tests a deliberately early and practically relevant recovery window, but it may also capture residual fatigue before enhancement is fully expressed. Evidence is especially scarce in adolescent athletes, in whom biological maturation and training history may limit the direct applicability of findings obtained in adult or elite populations [11,12]. Furthermore, countermovement-jump and change-of-direction performance differ across age categories in elite youth basketball players, reinforcing the need to investigate acute responses within specific developmental stages [13].

Previous acute plyometric studies have used heterogeneous conditioning volumes, including protocols ranging from 3 × 3 jumps to approximately 30 contacts, with responses varying according to exercise modality, task specificity, recovery duration, and participant characteristics [14,15]. However, there is currently insufficient evidence to determine the most appropriate dose of pogo-jump conditioning activity for eliciting PAPE, particularly in adolescent athletes. Consequently, it remains uncertain whether a brief, low-volume protocol of 24 bilateral pogo-jump contacts provides a sufficient conditioning stimulus to modify subsequent CMJ or DJ performance.

Accordingly, the primary objective of this randomized crossover pilot study was to obtain a preliminary estimate of the effect of adding 3 sets of 8 bilateral pogo jumps to a standardized warm-up, compared with time-matched low-intensity walking, on CMJ and DJ height during a sequential early post-conditioning assessment initiated 1 min after each condition. Secondary objectives were to document protocol completion, perceived exertion, and immediate post-protocol tolerability.

## 2. Materials and Methods

### 2.1. Study Design and Setting

This was a randomized, counterbalanced, within-participant crossover pilot study conducted at Club Baloncesto Moncada, Spain, between April and May 2026. Each participant completed an experimental pogo-jump condition and a low-intensity control condition, separated by a 7-day washout. Baseline anthropometry and familiarization were completed during an initial visit; the two experimental periods were conducted during the subsequent two weeks, in the usual club environment, between 18:00 and 20:00. Testing was scheduled at approximately the same time of day for each participant across periods.

Participants were allocated to one of two treatment sequences using sealed-envelope allocation: control followed by pogo jumps (sequence 1) or pogo jumps followed by control (sequence 2). Within each pair of participants, one envelope contained each sequence; the envelopes were closed, mixed, and selected without knowledge of their contents. The envelope was opened only after enrolment and completion of the baseline assessment, thereby concealing the upcoming sequence until assignment. Among the 21 completers, 11 were allocated to control–pogo and 10 to pogo–control. Owing to the visible nature of the interventions, participants and the evaluator supervising the sessions were not blinded after allocation; however, the sequence remained concealed until the envelope was opened.

The study is reported in accordance with the CONSORT extension for randomized crossover trials and, where applicable, the CONSORT extension for randomized pilot and feasibility trials [16,17]; the completed CONSORT checklist is provided as Supplementary File S1. The study was approved by the Biomedical Research Ethics Committee of Universidad Cardenal Herrera-CEU (reference CEEI26/816). Participants aged 18 years provided written informed consent. For participants younger than 18 years, written informed consent was obtained from a parent or legal guardian and assent was obtained from the participant. Procedures were conducted in accordance with the Declaration of Helsinki [18]. The study was registered at ClinicalTrials.gov (NCT07722273).

### 2.2. Participants

Male federation-registered basketball players aged 15–18 years were recruited by convenience sampling from the club’s cadet and junior teams. Eligibility required regular structured basketball training, the ability to understand the study instructions, safe execution of the jump tests after familiarization, written informed consent from participants aged 18 years or from a parent or legal guardian for participants younger than 18 years, and assent from minors. Exclusion criteria were an acute injury limiting participation, lower-limb pain aggravated by jumping, recent surgery or a relevant health condition, or inability to perform the test technique safely.

### 2.3. Sample Size

The sample-size calculation was reconstructed in G*Power 3.1 (Heinrich Heine University Düsseldorf, Düsseldorf, Germany) for the within-participant condition-by-time interaction. The source study, which examined the acute effect of a plyometric conditioning stimulus on CMJ performance, reported a standardized mean difference of d = 0.72 [7]. For a two-level repeated-measures contrast, this was converted using the assumed within-participant correlation (r = 0.50): d_z = d/√[2(1 − r)] = 0.72, corresponding to an interaction effect size f = d_z/2 = 0.36. The calculation used a two-sided α of 0.05, 80% power, one group, four repeated measurements (two conditions × two times), an assumed correlation of 0.50 among repeated measurements, and a non-sphericity correction of 1.00, yielding a minimum target of 15 completers. Given the pilot nature of the study, the sample-size calculation should not be interpreted as providing sufficient precision for a definitive efficacy conclusion. Accordingly, the revised interpretation prioritizes effect estimates and 95% confidence intervals.

### 2.4. Experimental Procedures

Each visit followed the same standardized sequence. Participants first completed the standardized team warm-up used for both periods, including low-intensity locomotion, dynamic lower-limb mobility, and progressive submaximal jump familiarization. They then performed three maximal CMJs followed by three DJs, with 30 s of passive recovery between jump types. Following completion of each condition, participants recovered for 1 min before beginning the post-conditioning jump assessments. Participants then performed three consecutive CMJs, followed by a predefined 30 s transition, and three consecutive DJs. The CMJ and DJ trials each required approximately 10–15 s to complete. Consequently, the complete post-conditioning battery was initiated 1 min after the intervention and finished within approximately 2 min after each condition. The highest valid trial was retained for each jump type. Thus, the 1 min interval referred to initiation of the assessment battery rather than to the exact timing of each trial or the retained maximum.

Participants used the same usual athletic footwear across visits. A trial was repeated when the participant removed the hands from the hips, used an additional preparatory step or hop, actively jumped from rather than stepped off the DJ box, failed to land bilaterally on the platform, lost balance on landing, or did not follow the prescribed CMJ or DJ technique. For both CMJ and DJ, the highest valid jump height from the three trials was retained for analysis.

To reduce acute performance-related confounding, participants were tested between 18:00 and 20:00 and at approximately the same clock time in both periods. They were instructed not to complete basketball practice, resistance exercise, plyometric exercise, or other vigorous training on the testing day; to avoid caffeine and other stimulants before testing; and to maintain their usual food and hydration habits without consuming a large meal immediately before the session. The coaching staff confirmed that no scheduled team training occurred before testing, and participants verbally confirmed compliance at each visit.

### 2.5. Interventions

Pogo-jump condition: Participants performed 3 sets of 8 consecutive bilateral ankle-dominant hops (24 contacts total), with 1 min of passive recovery between sets. They were instructed to jump with maximal intent, minimize ground-contact time, and maintain relative knee extension without a deep countermovement. The evaluator provided the standardized cue: “Touch and leave the ground quickly, like a spring”. Participants practiced the prescribed pogo-jump technique during familiarization, and an investigator visually monitored execution during the experimental sessions. However, this supervision ensured procedural compliance only and did not provide an objective quantification of the mechanical stimulus. The 3 × 8-repetition volume was selected pragmatically as a brief, low-volume microdose intended to provide repeated ankle-dominant stretch-shortening-cycle contacts while limiting protocol duration and the potential accumulation of fatigue. This volume was considered exploratory and not an established or optimal dose for eliciting PAPE.

Control condition: Participants walked continuously at a comfortable, low intensity for approximately 3 min, without running, jumping, or abrupt changes in pace. The duration was selected to approximate that of the experimental condition.

### 2.6. Outcomes and Instrumentation

The co-primary performance outcomes were CMJ height and DJ height (cm). Flight time was recorded using the Chronojump-Boscosystem contact platform (Chronojump, Barcelona, Spain) and converted to jump height [19]. For the CMJ, participants stood with hands on hips and performed a self-selected countermovement followed by a maximal vertical jump. For the DJ, participants stepped from an 18 cm box, landed bilaterally, and immediately jumped as high as possible while minimizing contact time. Three valid trials were completed at each time point. The highest valid height for each jump type was retained for the performance analysis. The highest valid trial was retained to represent maximal jump performance, whereas reliability across the three individual trials was analyzed separately and should not be interpreted as the reliability of the maximum-of-three endpoint.

Descriptive variables included age, standing height, body mass, body mass index, sitting height, estimated leg length, and standing vertical reach. Standing vertical reach was collected for descriptive purposes and was not included in inferential analyses. Anterior-knee pain was recorded immediately after the pogo-jump condition on a 0–10 numerical rating scale for descriptive tolerability monitoring, and perceived exertion after pogo jumps was assessed using the Borg 6–20 scale [20]. Protocol completion and adverse events were recorded by the evaluator. Biological maturation was described using maturity offset, expressed as the estimated number of years before or after peak height velocity and calculated from chronological age and anthropometric measurements using the sex-specific Mirwald equation [21]. Given the uncertainty of individual maturity-offset estimates, this variable was used descriptively rather than as a precise measure of biological age. The percentage of predicted adult height attained was estimated using the Khamis–Roche method [22]. Maturation variables were not included as covariates in the primary analysis.

### 2.7. Statistical Analysis

Analyses were conducted in IBM SPSS Statistics version 25 (IBM Corp., Armonk, NY, USA) using complete cases who completed both crossover periods. Continuous data are presented as mean ± standard deviation and range; categorical data are presented as *n* (%). Separate linear mixed-effects models were fitted for CMJ and DJ height. Condition, time, period, randomized sequence, the Condition × Time interaction, and the Period × Time interaction were included as fixed effects, with participant included as a random intercept. Time was categorized as PRE or POST + 1 min. The Condition × Time interaction represented the period-adjusted between-condition difference in change and was considered the primary treatment estimate. Adjusted estimates, 95% confidence intervals, and two-sided *p* values are reported. Consistent with the exploratory pilot design, interpretation focused primarily on the magnitude and 95% confidence interval of the period-adjusted treatment estimate, with *p* values considered secondary (statistical significance was set at *p* < 0.05).

A two-factor repeated-measures ANOVA, including Time and Condition as within-participant factors, was retained as a sensitivity analysis. F statistics, two-sided *p* values, and partial eta squared (η^2^p) are reported for this analysis. Because the CMJ change-score contrast showed marked non-normality and an influential observation, an additional sensitivity analysis used the Wilcoxon signed-rank test on the within-participant difference in change scores, defined as [(POST − PRE)pogo − (POST − PRE)control]. The same non-parametric analysis was applied to DJ for consistency. Potential residual carryover was explored by comparing period-2 PRE values between randomized sequence groups.

Relative reliability across the three trials at each condition and time point was quantified using a two-way mixed-effects, consistency, single-measure intraclass correlation coefficient (ICC(C,1)) with a 95% CI obtained by participant-level bootstrap resampling. ICC(C,1) was selected to evaluate the consistency of participants’ relative ranking across repeated trials, whereas absolute measurement error was evaluated separately using SEM and MDC95. ICC values were interpreted as <0.50 poor, 0.50–0.75 moderate, 0.75–0.90 good, and >0.90 excellent [23]. Absolute reliability was estimated as SEM = SD × √(1 − ICC), and MDC95 = 1.96 × √2 × SEM. SEM was calculated using an SD corresponding to the same single-trial measurement level as the ICC.

## 3. Results

### 3.1. Participant Flow and Characteristics

**Figure 1:**
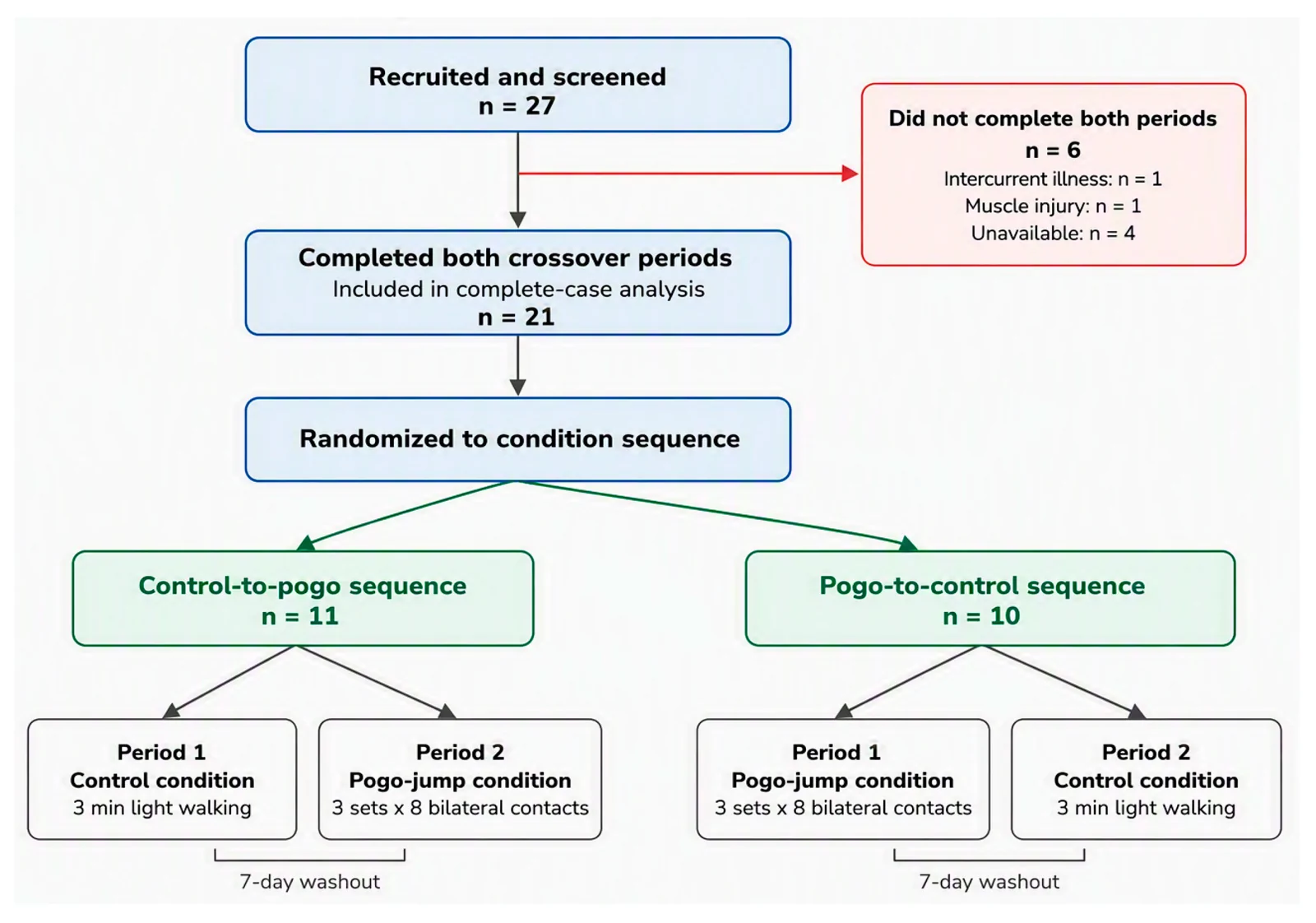
Participant flow through the randomized crossover pilot study.

Twenty-one of 27 recruited players completed both crossover periods and were included in the complete-case analysis (77.8% retention). Among completers, 11 participants followed the control–pogo sequence and 10 followed the pogo–control sequence. The pogo-jump condition consisted of 3 sets × 8 bilateral contacts, and the control condition consisted of 3 min of light walking. Participant flow is shown in Figure 1. Baseline characteristics are summarized in Table 1.

The estimated maturity offset was 1.51 ± 0.82 years, and all participants had an estimated positive maturity offset. The mean percentage of predicted adult height attained was 98.34 ± 1.65% (range, 95.24–100.54%). These estimates descriptively suggest that the sample was predominantly post-peak height velocity.

Ten of the 21 participants (47.6%) reported anterior-knee symptoms rated between 1 and 3 on the 0–10 numerical rating scale during the preceding week. This descriptive symptom frequency was not a prespecified comparative outcome and was not based on a clinical diagnosis of patellofemoral pain.

### 3.2. Jump Performance

As shown in Table 2, the period-adjusted between-condition difference in change was −0.89 cm for CMJ (95% CI, −2.92 to 1.14; *p* = 0.389) and 0.34 cm for DJ (95% CI, −1.77 to 2.45; *p* = 0.753). Both confidence intervals included values compatible with potentially meaningful effects in either direction. The Period × Time interaction was −0.57 cm for CMJ (95% CI, −2.60 to 1.45; *p* = 0.578) and 1.68 cm for DJ (95% CI, −0.43 to 3.79; *p* = 0.119). No sequence effect was identified for CMJ (*p* = 0.614) or DJ (*p* = 0.774).

The repeated-measures ANOVA sensitivity analysis yielded Time × Condition *p* values of 0.369 for CMJ and 0.577 for DJ, with partial eta squared values of 0.041 and 0.016, respectively. The CMJ change-score contrast was non-normally distributed (Shapiro–Wilk *p* < 0.001), whereas the DJ contrast was compatible with normality (*p* = 0.139). Wilcoxon sensitivity analyses yielded the same overall interpretation for CMJ (*p* = 0.658) and DJ (*p* = 0.320).

In the primary mixed-effects model, the Period × Time estimate for DJ indicated that the PRE-to-POST change was 1.68 cm greater in the second than in the first period; however, the confidence interval was wide and included zero (95% CI, −0.43 to 3.79; *p* = 0.119). Period-2 PRE values did not differ between sequence groups for CMJ (*p* = 0.580) or DJ (*p* = 0.553), providing no baseline evidence of material residual carryover.

CMJ within-session reliability (Table 3) was excellent at all assessment points, with ICC values ranging from 0.947 to 0.973. The corresponding SEM ranged from 1.14 to 1.57 cm and MDC95 from 3.16 to 4.35 cm. DJ reliability was moderate in control condition at PRE and POST + 1 min (ICC 0.669 and 0.652, respectively), excellent at intervention PRE (ICC 0.919), and good at intervention POST + 1 min (ICC 0.870). For DJ, SEM ranged from 1.70 to 3.03 cm and MDC95 from 4.72 to 8.39 cm.

**Figure 2:**
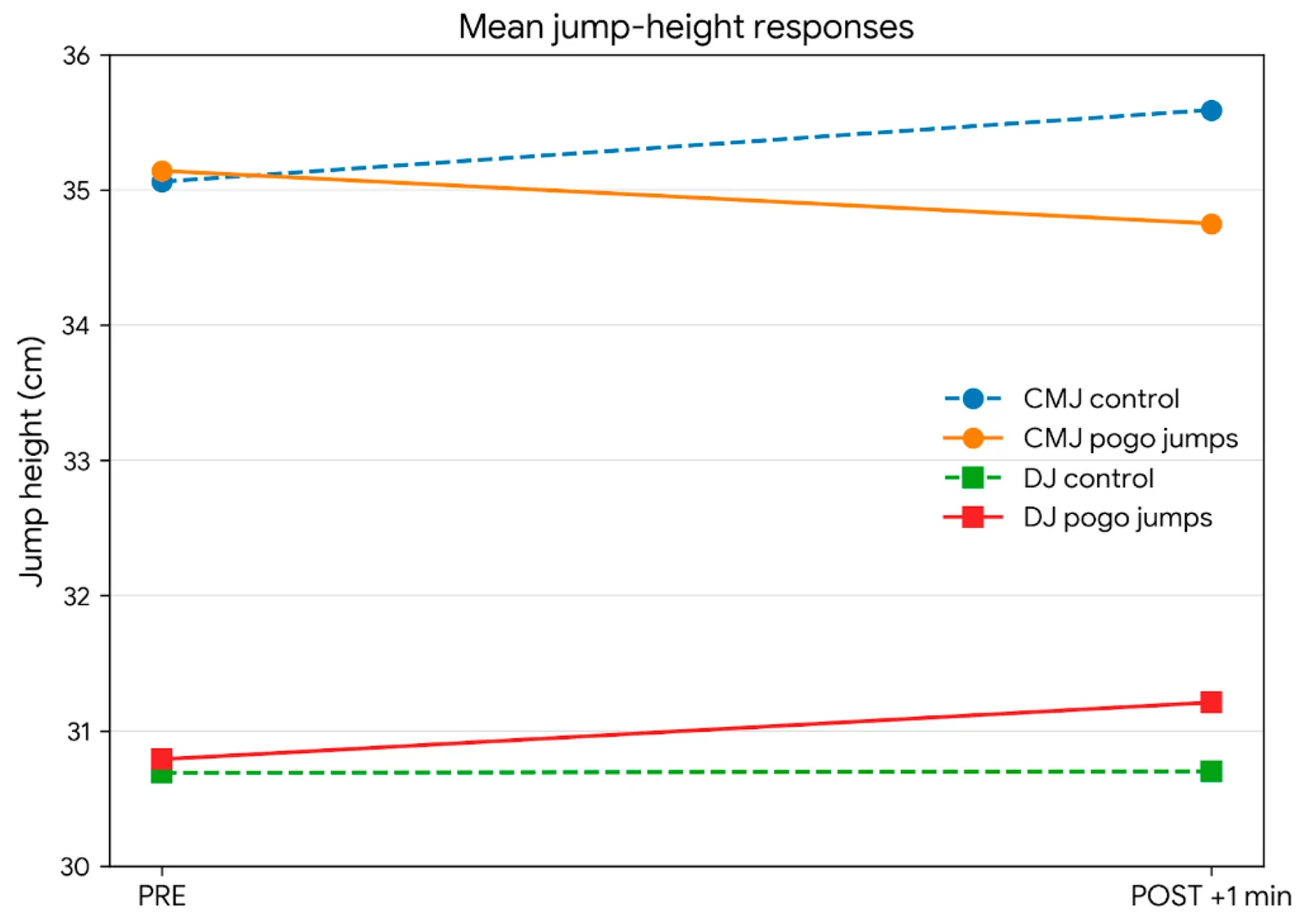
Mean CMJ and DJ height before and after the pogo-jump and control conditions.

Mean CMJ and DJ heights before and after the pogo-jump and control conditions are illustrated in Figure 2.

### 3.3. Protocol Completion, Perceived Exertion, and Immediate Tolerability

All 21 study completers performed the full pogo-jump protocol, corresponding to 100% protocol completion among completers. Mean perceived exertion was 11.4 ± 1.2 on the Borg 6–20 scale, corresponding to a light-to-moderate effort. Immediately after the pogo-jump condition, all completers reported 0/10 anterior-knee pain, and no immediate adverse event was documented. After the pogo-jump condition, 9 of 21 participants (42.9%) rated their jump performance as “somewhat better,” whereas 12 (57.1%) rated it as unchanged; no participant rated it as worse.

## 4. Discussion

The main finding was that adding 3 sets of 8 bilateral pogo jumps to a standardized warm-up did not produce a detectable group-level improvement in CMJ or DJ height during the early post-conditioning assessment beginning 1 min after each condition. The period-adjusted between-condition difference in change was −0.89 cm for CMJ and 0.34 cm for DJ. The corresponding 95% confidence intervals included zero and were compatible with potentially meaningful effects in either direction. Therefore, this pilot study did not estimate the effects with sufficient precision to exclude either a beneficial or detrimental response. The repeated-measures and non-parametric sensitivity analyses yielded the same overall interpretation, indicating that explicit adjustment for period and randomized sequence did not materially change the direction or interpretation of the treatment estimates. Consequently, the findings should be interpreted cautiously and should not be considered evidence of equivalence or absence of an effect. 

All participants completed the same familiarization procedures, standardized warm-up, and baseline jump assessments in both experimental conditions. The warm-up was included to ensure adequate and safe preparation for the conditioning activity and subsequent CMJ and DJ assessments. Therefore, familiarization and warm-up were not differential components between conditions. Following the warm-up and baseline measurements, the only experimental difference was the allocated condition: 3 × 8 bilateral pogo jumps or time-matched low-intensity walking. Accordingly, the study evaluated the incremental effect of adding a pogo-jump microdose to an already completed standardized warm-up rather than the effect of pogo jumps relative to rest.

The absence of a detectable immediate improvement is broadly compatible with previous research examining acute neuromuscular interventions in youth basketball players. Sos-Tirado et al. reported acute improvements in hamstring flexibility after proprioceptive neuromuscular facilitation, performed alone or combined with neuromuscular electrical stimulation, without a concomitant change in CMJ performance [24]. Although the intervention and primary outcomes differed, both studies indicate that a brief neuromuscular intervention may modify a specific physical characteristic without necessarily improving vertical-jump height immediately.

Performance following a conditioning activity reflects the balance between performance-enhancing and fatiguing processes and depends on the activity type, volume, recovery interval, training status, and performance task [4,5,6,9,10,25]. One possible explanation is that the 1 min interval did not coincide with the optimal response window. Although the complete battery was finished within approximately 2 min after each condition, the CMJ trials preceded the DJ trials and the highest valid trial was retained. Consequently, the present findings characterize performance during a short early post-conditioning window but cannot isolate the response at exactly 1 min or determine the subsequent time course of PAPE. Residual fatigue may have offset a potentiating effect, although fatigue was not measured directly. Alternatively, the conditioning activity may not have provided a sufficient stimulus under the conditions tested. Barreto et al. observed CMJ enhancement across several post-conditioning time points after a higher-volume plyometric activity in active adults [8], but differences in participants, exercise dose, comparator, and repeated testing preclude direct extrapolation. Similarly, Papale et al. reported heterogeneous acute CMJ responses following rebound-type exercise, suggesting that the balance between facilitation and fatigue may vary according to participants’ baseline characteristics [26]. Because only one dose and one post-conditioning time point were examined, the present study cannot distinguish among these explanations.

The magnitude of the conditioning stimulus is another consideration. The protocol elicited a mean perceived exertion of 11.4 on the Borg 6–20 scale, indicating a relatively low perceived internal load. However, perceived exertion does not directly quantify the mechanical or neuromuscular stimulus, and pogo-jump height, ground-contact time, and joint kinetics were not recorded. Habitual plyometric exposure and recent training load were also not quantified. It therefore cannot be concluded that 24 contacts were insufficient or that a larger dose would have produced a greater response; increasing the dose could enhance either potentiation or fatigue.

Biological maturation should be considered when defining the population to which the findings apply. All participants had positive estimated maturity-offset values and had attained more than 95% of their predicted adult height. Previous research in elite youth basketball players has identified age- and sex-related differences in CMJ and change-of-direction performance and associations between physical performance and ultrasound-derived lower-limb tissue characteristics [13]. Nevertheless, maturity offset and predicted adult height are indirect anthropometric estimates subject to prediction error. Maturation was used descriptively, and the pilot sample was not designed to determine whether maturity status modified the acute response [11,12]. The findings are therefore most directly applicable to relatively mature adolescent male basketball players and should not be generalized to younger, less mature, or female populations.

Interpretation of the DJ estimate requires particular caution. CMJ reliability was excellent, whereas DJ reliability ranged from moderate to excellent across assessment points. The greater coordinative demands of the DJ and possible remaining familiarization effects may have contributed to this variability, further increasing the uncertainty of the DJ treatment estimate. Although DJ height increased descriptively by 0.42 cm after the pogo-jump condition and remained essentially unchanged after control, the period-adjusted treatment estimate was small and imprecise, and the MDC95 estimates were substantially larger than the observed mean change. The Period × Time estimate suggested possible period-related variation in DJ change, but its confidence interval was wide and included zero. Together with the small pilot sample, and the possibility of incomplete familiarization, these factors substantially increase uncertainty around the DJ treatment estimate. Therefore, the descriptive change in DJ height should not be attributed confidently to the pogo-jump condition or used to support a mechanistic interpretation. CMJ demonstrated better within-session reliability; despite marked non-normality and an influential observation in its change-score contrast, the Wilcoxon sensitivity analysis yielded the same overall interpretation as the primary mixed-effects model.

Protocol completion and immediate tolerability were favorable. All participants who completed both periods performed the full pogo-jump protocol, perceived exertion was light to moderate, and none reported anterior-knee pain immediately afterward. However, symptoms reported during the preceding week were self-reported and were not based on a clinical diagnosis of patellofemoral pain. Delayed pain, tendon symptoms, landing mechanics, cumulative mechanical load, training availability, and adverse events beyond the immediate assessment were not comprehensively evaluated. These findings therefore support favorable immediate tolerability but do not establish musculoskeletal safety, injury-prevention effectiveness, or long-term acceptability.

The randomized crossover design increased analytical efficiency by allowing each participant to serve as his own control. Additional strengths included concealed sequence allocation, a time-matched control condition, standardized procedures, consistent testing times, a period-adjusted primary analysis, and complementary sensitivity analyses. The field-based setting enhanced ecological validity and demonstrated practical protocol delivery, while the findings may inform the timing and dosing of future PAPE protocols in youth basketball.

Several limitations should be considered when interpreting these findings. The study included a small convenience sample of male players from a single club, and the analysis was restricted to participants who completed both experimental periods. Because baseline information was incomplete for non-completers, formal comparisons between completers and non-completers were not possible, and attrition bias cannot be excluded. Anterior-knee pain was not assessed immediately before the intervention or before and after the control condition. Therefore, the post-pogo pain score cannot be interpreted as a condition-specific change and provides only limited descriptive information regarding immediate tolerability. The anterior-knee symptoms reported during the preceding week were self-reported and were not based on a clinical diagnosis of patellofemoral pain. Furthermore, owing to the visible differences between conditions, neither participants nor the session evaluator could be blinded. The conditioning stimulus was not mechanically quantified; therefore, potential between-participant differences in pogo-jump height, ground-contact time, reactive strength index, lower-limb stiffness, or performance decrement could not be evaluated. The assessment began 1 min after each condition and was completed within approximately 2 min. As exact trial timestamps were unavailable, the outcomes could not be assigned to a precise post-conditioning time point. Future randomized crossover studies should include larger and more diverse samples, use prospectively registered protocols and time-stamped statistical analysis plans, compare multiple pogo-jump doses, and assess performance across several post-conditioning intervals. More extensive DJ familiarization and adequately powered crossover analyses incorporating treatment, period, sequence, and period-related changes are also warranted. Future research should quantify recent training load and recovery status, as well as pogo-jump height and contact time, and should evaluate individual response distributions, reactive strength index, and force–time variables alongside jump height. This approach would help determine whether maturation, sex, training age, and habitual plyometric exposure influence the response.

## 5. Conclusions

In adolescent male basketball players, adding a microdose of 24 bilateral pogo jumps to a standardized warm-up did not produce a detectable group-level improvement in CMJ or DJ height relative to low-intensity walking during the early post-conditioning assessment beginning 1 min after each condition. However, the confidence intervals remained compatible with potentially meaningful effects in either direction. The protocol was completed by all study completers and showed favorable immediate tolerability. The small pilot sample, single dose, single early assessment window, and variable DJ reliability limit the precision and generalizability of the findings. The study does not determine whether a different dose or longer recovery interval could produce PAPE and does not establish long-term safety or injury-prevention effectiveness. These findings should not be interpreted as demonstrating equivalence or absence of an effect.

Practical Applications: Practitioners should not expect a consistent group-level improvement in CMJ or DJ height after adding 3 sets of 8 bilateral pogo jumps to a standardized warm-up in adolescent male basketball players. The protocol was brief, required no external load, was completed by all study completers, and elicited light-to-moderate perceived exertion, with no anterior-knee pain reported immediately after the pogo-jump condition. The present findings do not support using this specific protocol to enhance jump height during the early post-conditioning assessment beginning 1 min after the condition. Other doses and recovery intervals require direct evaluation, and the protocol should not replace a comprehensive warm-up or be promoted as an injury-prevention strategy.

## Figures and Tables

**Table 1:** Baseline characteristics of the analyzed sample (*n* = 21).

Variable	Mean ± SD	Minimum	Maximum
Age (years)	16.55 ± 0.88	15.37	18.21
Standing height (cm)	177.22 ± 6.05	163.90	190.00
Body mass (kg)	71.04 ± 11.03	59.00	101.00
Body mass index (kg/m^2^)	22.56 ± 2.76	18.99	31.17
Sitting height (cm)	91.70 ± 3.05	85.70	97.10
Estimated leg length (cm)	85.52 ± 4.40	76.00	92.90
Field vertical-reach measure (cm)	289.23 ± 14.04	258.8	309.00
Maturity offset (years from PHV)	1.51 ± 0.82	0.13	3.13
Predicted adult height attained (%)	98.34 ± 1.65	95.24	100.54

SD, standard deviation; PHV, peak height velocity. Standing vertical reach was collected for descriptive purposes and was not included in inferential analyses. Predicted adult height values slightly above 100% may occur because of prediction error.

**Table 2:** Jump-height outcomes before and after each condition (*n* = 21).

Outcome	Condition	PRE, Mean ± SD (Range), cm	POST + 1 min, Mean ± SD (Range), cm	Mean Change, cm	Period-Adjusted Between- Condition Difference in Change (95% CI), cm; *p* Value
CMJ	Pogo jumps	35.14 ± 5.90 (22.65–46.31)	34.75 ± 5.63 (22.09–45.58)	−0.39	−0.89 (−2.92 to 1.14); *p* = 0.389
CMJ	Control	35.06 ± 5.23 (24.93–46.60)	35.59 ± 7.28 (21.07–55.55)	0.53	
DJ	Pogo jumps	30.79 ± 6.14 (16.52–41.68)	31.21 ± 4.69 (21.43–39.70)	0.42	0.34 (−1.77 to 2.45); *p* = 0.753
DJ	Control	30.69 ± 5.64 (21.02–41.68)	30.70 ± 5.27 (21.63–41.25)	0.00	

CMJ: countermovement jump; DJ: drop jump; SD: standard deviation; CI: confidence interval.

**Table 3:** Within-session relative and absolute reliability of the three CMJ and DJ trials.

Outcome	Condition	Time	ICC	95% CI	SEM (cm)	MDC95 (cm)
CMJ	Control	PRE	0.964	0.924–0.987	1.33	3.69
CMJ	Control	POST + 1 min	0.947	0.852–0.981	1.57	4.35
CMJ	Pogo jumps	PRE	0.973	0.921–0.988	1.14	3.16
CMJ	Pogo jumps	POST + 1 min	0.952	0.780–0.991	1.52	4.22
DJ	Control	PRE	0.669	0.240–0.946	3.03	8.39
DJ	Control	POST + 1 min	0.652	0.297–0.928	2.96	8.21
DJ	Pogo jumps	PRE	0.919	0.812–0.961	1.70	4.72
DJ	Pogo jumps	POST + 1 min	0.870	0.696–0.950	1.79	4.97

ICC: intraclass correlation coefficient; CI: confidence interval; SEM: standard error of measurement; MDC95: minimal detectable change at 95% confidence. POST + 1 min: sequential early post-conditioning assessment beginning 1 min after each condition and completed within approximately 2 min.

## Data Availability

The data supporting the findings of this study are available from the corresponding author upon reasonable request. The data are not publicly available because they include information from adolescent participants and are subject to ethical and privacy restrictions.

## Supplementary Materials

The following supporting information can be downloaded at https://www.mdpi.com/article/10.3390/jfmk11030366/s1. Supplementary File S1: Completed CONSORT checklist for randomized crossover and pilot trials.

## References

[1] Komi P.V. (2000). Stretch-shortening cycle: A powerful model to study normal and fatigued muscle. J. Biomech..

[2] Nicol C., Avela J., Komi P.V. (2006). The stretch-shortening cycle: A model to study naturally occurring neuromuscular fatigue. Sports Med..

[3] Markwick W.J., Bird S.P., Tufano J.J., Seitz L.B., Haff G.G. (2015). The intraday reliability of the reactive strength index calculated from a drop jump in professional men’s basketball. Int. J. Sports Physiol. Perform..

[4] Blazevich A.J., Babault N. (2019). Post-activation potentiation versus post-activation performance enhancement in humans: Historical perspective, underlying mechanisms, and current issues. Front. Physiol..

[5] Xu K., Blazevich A.J., Boullosa D., Ramírez-Campillo R., Yin M.Y., Zhong Y.M., Tian Y.H., Finlay M., Byrne P.J., Cuenca-Fernández F. (2025). Optimizing post-activation performance enhancement in athletic tasks: A systematic review with meta-analysis for prescription variables and research methods. Sports Med..

[6] Zheng Z., Gao X., Song Z. (2026). Effects of post-activation performance enhancement added to general warm-up on jump, sprint, and change-of-direction performance in competitive athletes: A systematic review and meta-analysis. BMC Sports Sci. Med. Rehabil..

[7] Tobin D.P., Delahunt E. (2014). The acute effect of a plyometric stimulus on jump performance in professional rugby players. J. Strength Cond. Res..

[8] Barreto M.V.C., Telles J.F.S., de Castro M.R., Mendes T.T., Rodrigues C.P., de Freitas V.H. (2023). Temporal response of post-activation performance enhancement induced by a plyometric conditioning activity. Front. Sports Act. Living.

[9] Seitz L.B., Haff G.G. (2016). Factors modulating post-activation potentiation of jump, sprint, throw, and upper-body ballistic performances: A systematic review with meta-analysis. Sports Med..

[10] Gouvêa A.L., Fernandes I.A., César E.P., Silva W.A.B., Gomes P.S.C. (2013). The effects of rest intervals on jumping performance: A meta-analysis on post-activation potentiation studies. J. Sports Sci..

[11] Lloyd R.S., Oliver J.L., Faigenbaum A.D., Myer G.D., De Ste Croix M.B.A. (2014). Chronological age vs. biological maturation: Implications for exercise programming in youth. J. Strength Cond. Res..

[12] Moran J., Sandercock G.R.H., Ramírez-Campillo R., Todd O., Collison J., Parry D.A. (2017). Maturation-related effect of low-dose plyometric training on performance in youth hockey players. Pediatr. Exerc. Sci..

[13] Lisón J.F., García-Herreros S., Ricart B., Godoy E.J., Nozal S., Cotolí-Suarez P., Jordán-López J., Amer-Cuenca J.J., Salvador-Coloma P. (2023). Ultrasound measurements and physical fitness of elite youth basketball players. Int. J. Sports Med..

[14] Werfelli H., Hammami R., Selmi M.A., Selmi W., Gabrilo G., Clark C.C.T., Duncan M., Sekulic D., Granacher U., Rebai H. (2021). Acute effects of different plyometric and strength exercises on balance performance in youth weightlifters. Front. Physiol..

[15] Zisi M., Stavridis I., Bogdanis G., Terzis G., Paradisis G. (2023). The acute effects of plyometric exercises on sprint performance and kinematics. Physiologia.

[16] Dwan K., Li T., Altman D.G., Elbourne D. (2019). CONSORT 2010 statement: Extension to randomised crossover trials. BMJ.

[17] Eldridge S.M., Chan C.L., Campbell M.J., Bond C.M., Hopewell S., Thabane L., Lancaster G.A. (2016). CONSORT 2010 statement: Extension to randomised pilot and feasibility trials. BMJ.

[18] World Medical Association WMA Declaration of Helsinki: Ethical Principles for Medical Research Involving Human Participants. https://www.wma.net/policies-post/wma-declaration-of-helsinki/.

[19] De Blas X., Padullés J.M., López del Amo J.L., Guerra-Balic M. (2012). Creation and validation of Chronojump-Boscosystem: A free tool to measure vertical jumps. RICYDE Rev. Int. Cienc. Deporte.

[20] Borg G. (1998). Borg’s Perceived Exertion and Pain Scales.

[21] Mirwald R.L., Baxter-Jones A.D.G., Bailey D.A., Beunen G.P. (2002). An assessment of maturity from anthropometric measurements. Med. Sci. Sports Exerc..

[22] Khamis H.J., Roche A.F. (1994). Predicting adult stature without using skeletal age: The Khamis–Roche method. Pediatrics.

[23] Koo T.K., Li M.Y. (2016). A guideline of selecting and reporting intraclass correlation coefficients for reliability research. J. Chiropr. Med..

[24] Sos-Tirado M., Campo-Manzanares A., Aguado-Oregui L., Cerdá-Calatayud C., Guardiola-Ruiz J.C., García-Lucas C., Montañez-Aguilera F.J., Lisón J.F., Amer-Cuenca J.J. (2024). Short term effects of proprioceptive neuromuscular facilitation combined with neuromuscular electrical stimulation in youth basketball players: A randomized controlled trial. J. Funct. Morphol. Kinesiol..

[25] Wilson J.M., Duncan N.M., Marin P.J., Brown L.E., Loenneke J.P., Wilson S.M.C., Jo E., Lowery R.P., Ugrinowitsch C. (2013). Meta-analysis of postactivation potentiation and power: Effects of conditioning activity, volume, gender, rest periods, and training status. J. Strength Cond. Res..

[26] Papale O., Festino E., De Maio M., Di Rocco F., Zema S., Cortis C., Fusco A. (2026). Acute effects of a mini-trampoline training session for improving normalized symmetry index in participants with higher baseline inter-limb asymmetry. Healthcare.

